# Secure Content Distribution with Access Control Enforcement in Named Data Networking

**DOI:** 10.3390/s21134477

**Published:** 2021-06-30

**Authors:** Htet Htet Hlaing, Yuki Funamoto, Masahiro Mambo

**Affiliations:** 1 Graduate School of Natural Science and Technology, Kanazawa University, Kanazawa 920-1192, Japan; yuki0323@stu.kanazawa-u.ac.jp; 2 Institute of Science and Engineering, Kanazawa University, Kanazawa 920-1192, Japan; mambo@ec.t.kanazawa-u.ac.jp

**Keywords:** named data networking (NDN), secure content distribution, content security, content confidentiality, encryption-based access control

## Abstract

NDN is one of the new emerging future internet architectures which brings up new solutions over today’s internet architecture, facilitating content distribution, in-network caching, mobility support, and multicast forwarding. NDNs ubiquitous in-network caching allows consumers to access data directly from the intermediate router’s cache. However, it opens content privacy problems since data packets replicated in the router are always accessible by every consumer. Sensitive contents in the routers should be protected and accessed only by authorized consumers. Although the content protection problem can be solved by applying an encryption-based access control policy, it still needs an efficient content distribution scheme with lower computational overhead and content retrieval time. We propose an efficient and secure content distribution (ES_CD), by combining symmetric encryption and identity-based proxy re-encryption. The analysis shows that our proposed scheme achieves content retrieval time reduction up to 20% for the cached contents in our network simulation environment and a slight computational overhead of less than 19 ms at the content producer and 9 ms at the consumer for 2 KB content. ES_CD provides content confidentiality and ensures only legitimate consumers can access the contents during a predefined time without requiring a trusted third party and keeping the content producer always online.

## 1. Introduction

With the growing number of Internet usage and content distribution services on today’s Internet, researchers proposed a better way to distribute the content using the name instead of using the address or location. Consequently, information-centric networking (ICN) becomes the ultimate future internet architecture to replace the host-centric TCP/IP architecture with content-based architecture. NDN is one of the dominant candidates in the ICN architecture in which contents become the priority elements to address the current internet architecture limitations [1,2].

One of the main features of NDN is in-network caching, where contents can be cached in the intermediate routers in the network. Since the cached contents are widely available at the intermediate routers, data access control turns out to be problematic [3]. The content producer may lose control over the contents which have been disseminated to the network. A malicious consumer can easily access the cached contents from the cache and attempt unauthorized decryption without the content producer’s permission. This issue has received substantial attention, and most of the research in the NDN field aims to solve it to protect the sensitive contents by using encryption-based access control technologies [4,5,6].

In the encryption-based access control scheme, sensitive contents are protected against unauthorized access by content encryption. The conventional end-to-end content encryption approaches provide flexible access control, i.e., specifying access rights for individual users. However, these approaches not only undermine in-network caching ability but also introduce a significant computational overhead and delay in NDN architecture. This is because contents are encrypted with different keys and are stored in the cache. Such encrypted contents are accessible only by the consumers with the corresponding decryption key [7], and the content producer faces the increase in computational overhead by generating a ciphertext for each requested consumer.

Although several approaches [4,5,6] have been proposed for content security and access control issues from different aspects, there are still some open challenges and problems to solve [8]. First of all, the in-network caching mechanism is not utilized, which cause content retrieval delay, and the content producer needs to be always online for the consumer authentication process and the decryption key request, which incurs additional communication delay in NDN [4]. Second, a trusted third party, such as trusted authority (TA) or access authority (AA), is needed in some constructions like [9] to manage the access policy and update keys, which may somehow leak the user’s confidential information to the adversary. Besides, some flexible access control schemes may incur the computational overhead for managing the key to protect each content since the consumer needs to contact the content producer for access right verification for every content request [5,6]. It is important to reduce the computational and communication overhead for key management to protect the content in NDN architecture [6,8,9,10].

Based on the above studies, we present an efficient and secure content distribution solution (ES_CD) for NDN architecture, which provides content confidentiality by encryption, as well as a limited access time for each consumer. To be specific, our design aims were: (1) to disseminate the encrypted content securely only to legitimate consumers confidentially and efficiently with fast content retrieval time, (2) to enforce flexible access control on the encrypted data by securely distributing the corresponding decryption key, and (3) to ensure the user revocation without requiring the contact to the content producer. Our scheme can guarantee that the content producer can handle all the accesses by defining the access time while all legitimate consumers can access the requested data through a nearby router to leverage the in-network caching mechanism. Our approach can be summarized as follows:

- We propose and implement an encryption-based access control mechanism to effectively protect sensitive content in the NDN network using symmetric encryption and identity-based proxy re-encryption (IB-PRE) scheme;
- We design and develop an efficient and secure content distribution scheme to prevent unauthorized consumers from obtaining sensitive and cached contents by setting predefined access time;
- We evaluate and demonstrate through experimental results that our scheme is applicable in NDN without undermining the core NDN architecture, achieves flexible access control, and introduces only a slight overhead on NDN end entities.

The remainder of this paper is organized as follows. In Section 2, we briefly explore background of NDN, access control mechanisms and basic cryptographic definitions and notations. Section 3 shows system overview and security assumptions, while Section 4 describes the details of our proposed mechanism. We evaluate our system in the NDN framework and show its related security and performance analysis in Section 5. In Section 6, we compare our scheme with some other previous works. Section 7 examines the related work and, lastly, we conclude the paper and describes the final remarks in Section 8.

## 2. Background

We provide a brief introduction to NDN concepts and discuss about the encryption-based access control in this section.

### 2.1. A Quick Recap of NDN Architecture

In NDN, communication is driven by a consumer by sending interest packets with the name of the data and receiving data packets from the content producer.

- Interest Packet: generated by the consumers, which brings a hierarchical name related to the target desired content data.
- Data Packet: produced by the content producer, which contains the combination of both the name and the requested content data.

NDN uses hierarchically structured names for routing and forwarding the packets, similar to URLs in today’s internet architecture. NDN names can be anything; for example, a chunk of a movie from Netflix can be represented, /netflix/movie/001. Hence, NDN names play the primary role in the NDN architecture for packet transmission, and they are independent of the location of the content or endpoints [11]. The built-in security of NDN can be guaranteed by signing every data packet securely at the content producer side. The fundamental NDN architecture consists of the following entities [2]:

- Consumer: who sends request interest packets to the content producer;
- Data Producer: who produces a data packet based on the consumer’s request and returns it to the consumer through the reverse path of the interest packet;
- Router: who forwards the interest and data packets to and from the content producer and the consumer; caches the returned data packet to satisfy the future requests from the consumer.

**Figure 1:**
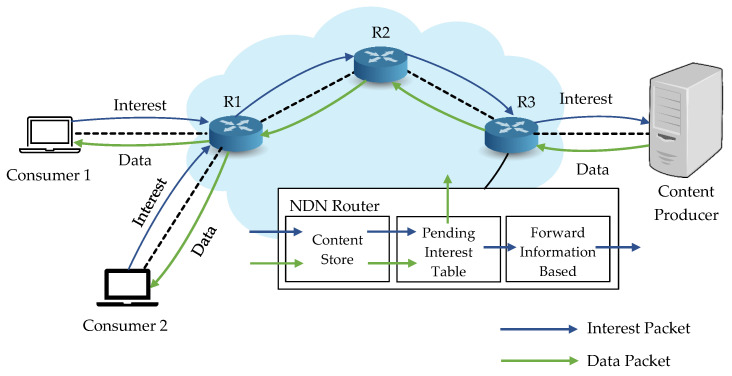
Fundamental architecture of NDN.

Every NDN router keeps three tables: content store (CS), pending interest table (PIT), and forward information base (FIB) to forward the packets. When an interest packet arrives at the NDN router, it first needs to check in the CS if any cached data matches the requested name. If there is a copy, it directly replies to the data back to the consumer. Otherwise, it will search in PIT to check the outgoing interface for the same content. If a match is found, it updates with the new interface, and the interest will be discarded. If not, it adds the interface to the PIT. The interest packet arrives at FIB, where the names and the corresponding interfaces are stored to forward to the content producer [1,12]. After all these processes, the data will be sent back to the requested consumer, as shown in Figure 1.

### 2.2. Encryption-Based Access Control and Challenges in NDN

**Figure 2:**
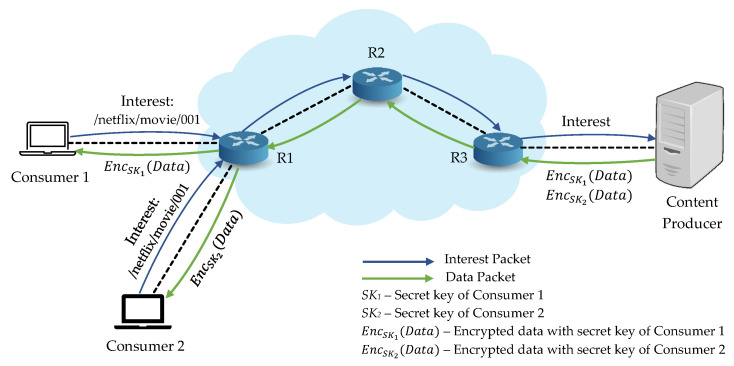
Encryption-based access control scheme in NDN.

As the main feature of NDN is in-network caching, contents may be cached in untrusted caches or easily retrieved by an unauthorized consumer and content security turns into a critical problem to resolve. Additionally, the basic security features of NDN cannot provide content security regarding confidentiality and access control. Especially, access control becomes a vital factor to limit content access to consumers [5,13]. To deal with content security issue, encrypting contents using modern encryption schemes is one of the best ways to guarantee secure content distribution and fine-grained access control [14,15], as illustrated in Figure 2. One of the challenges in designing a cryptosystem in NDN to encrypt the contents is how to use symmetric key scheme or public key encryption scheme. Since they have their advantages and limitations, one needs to consider the usage based on the system requirements, such as faster key generation, the complexity of managing access control, and computational cost. Nevertheless, combining these two cryptographic schemes can be considered to provide higher efficiency in terms of key generation and distribution in NDN [4,16].

In addition, NDN combined with cryptographic schemes can also lead to myriad problems, for example, key distribution, key/user revocation, communication, and computational overhead for encrypting every content [17]. The main problem of user revocation in NDN is that the revoked users can still access and decrypt cached data with the old keys. When revocation happens, the ciphertext needs to be updated into a new ciphertext and re-encrypted by the content producer with the new key. However, such an update leads to a high computational cost and old cached ciphertext unusable in NDN. Therefore, a new approach is needed to utilize cached contents efficiently and distribute them securely to the NDN network with the enforcement of an effective access control mechanism [7].

### 2.3. Proxy Re-Encryption-Based Access Control Schemes in NDN

Proxy re-encryption (PRE) [18,19] is an alternative answer that can provide comparable properties as the traditional end-to-end encryption scheme with more efficiency to ensure the security of sharing data and address the revocation issue in NDN. PRE can be applied in the NDN environment to implement an encryption-based access control mechanism, which generates re-encryption key to permit access right for the consumers and re-encrypts the content by the content producer or network routers [20,21].

**Figure 3:**
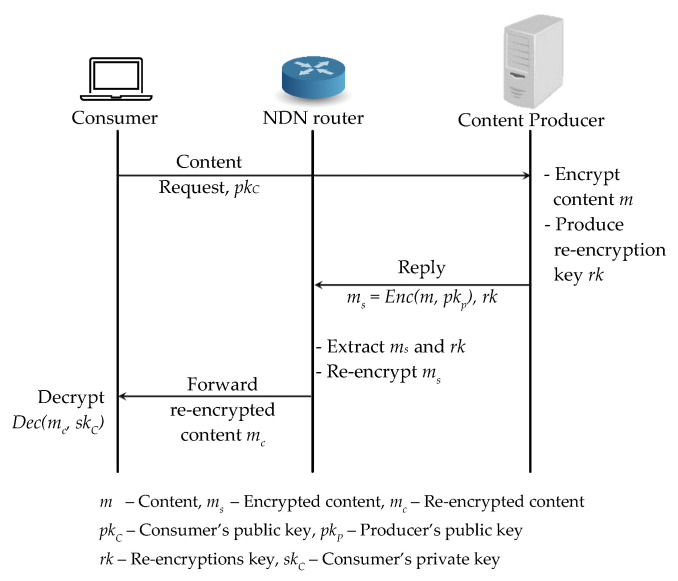
Basic Proxy Re-Encryption scheme application to NDN architecture.

We shows a PRE-based access control scheme in Figure 3 by applying the proxy re-encryption scheme to NDN in a straightforward way. When a content producer receives a content request from a consumer together with the consumer’s public key $pk_{C}$, it first encrypts the original content *m* with its public key and generates a re-encryption key rk. Then the content producer replies a first layer encrypted content $m_{s}$ and the re-encryption key rk to the consumer and it first arrives at the router. Then the router re-encrypts the first layer encrypted content $m_{s}$ to the second layer encrypted content $m_{c}$ by using rk and forwards the generated $m_{c}$ to the consumer. As soon as the consumer receives the second layer encrypted content $m_{c}$, it decrypts $m_{c}$ with its secret key $sk_{C}$ to access the original content *m*. Such a straightforward application of the PRE scheme to NDN needs to re-encrypt the first layer encrypted content $m_{s}$ and re-publish $m_{c}$ to the network for every requested consumer and such a $m_{c}$ can be decrypted by the corresponding requested consumer with $sk_{C}$. Other consumers cannot utilize the cached contents $m_{c}$ directly from the cache and NDNs in-network caching feature is not fully effective.

Identity-based Proxy Re-encryption (IB-PRE) is one of the main branches of PRE and considered as a good candidate for secure content distribution and access control enforcement in content delivery network architectures. The first IB-PRE scheme was proposed by Green and Ateniese in 2007 [22] and is based on the Boneh-Franklin’s identity-based encryption (IBE) scheme [23]. In IB-PRE, the semi-trusted proxy alters a first layer ciphertext encrypted by the sender’s identity into a second layer ciphertext by using the re-encryption key that can be finally decrypted by the receiver’s secret key related to its identity. IB-PRE can be used for different purposes in various practical applications, such as access control and key management in network file storage, secure access delegation in cloud storage, secure email systems, etc.

To enforce the encryption-based access control in NDN architecture, the content producer can encrypt the sensitive contents by adding some access control policies before dissemination, and network entities can perform as a semi-trusted proxy for re-encryption. Then the authenticated consumers with the access policy can access the decryption key and decrypt the ciphertext. Thus, it is suitable to insert the proxy to NDN by adding some features to the NDN entities without affecting the core NDN architecture [24].

### 2.4. Basic Definitions and Notations

We briefly examine basic security notions and the cryptographic primitives used in the construction of our proposed scheme.

#### 2.4.1. Cryptographic Background

**Definition** **1.** *(Bilinear maps) Let $G,G_{T}$ be two cyclic groups of the same prime order q, writing the group action multiplicatively. We assume that g is a generator of group G. Let e:G×G→$G_{T}$ be a bilinear map with the following properties:*

- *Bilinearity: $e\left(g_{1}^{a},g_{2}^{b}\right)=e{\left(g_{1},g_{2}\right)}^{a}$${}^{b}$ for all $g_{1},g_{2}\in G$, and $a,b\in_{R}Z_{q}$;*
- *Non-degeneracy: There exists $g_{1},g_{2}\in G$ with $e(g_{1},g_{2})\neq 1$, in other words, the map does not send all pairs in G×G to the identity in $G_{T}$;*
- *Computability: There is an efficient algorithm to compute $e(g_{1},g_{2})$ for all $e(g_{1},g_{2})\in G$.*

**Definition** **2.** *(DBDH problem) The Decisional Bilinear Diffie-Hellman (DBDH) problem is that, given a tuple of values $g,g^{a},g^{b},g^{c}\in G$ for random value $x,y,z\in_{R}Z_{q}$ and $T\in_{R}G_{T}$, to decide whether $T=e{\left(g,g\right)}^{abc}$. Let k be a security parameter of sufficient size. Formally, we say that the DBDH assumption holds in $(G,G_{T}$ if for all probabilistic polynomial time algorithms A, the following condition is true:*

(1) $$\left|\begin{array}{c} P_{r}\left[\begin{array}{c} a,b,c\overset{\$}{\leftarrow }Z_{q}^{*};1\leftarrow A\left(g,g^{a},g^{b},g^{c},e{\left(g,g\right)}^{a}bc\right). \end{array}\right]-\\ P_{r}\left[a,b,c\overset{\$}{\leftarrow }Z_{q}^{*};T\overset{\$}{\leftarrow }G_{T};1\leftarrow A\left(g,g^{a},g^{b},g^{c},T\right).\right] \end{array}\right|\leq v(k)$$

*where v(.) is defined as a negligible function, i.e., for all polynomial functions p(.), there exists l such that for all k > l, v(k)<1/p(k) [22].*

#### 2.4.2. Symmetric Key Encryption

Symmetric key encryption uses the same key for both sender and receiver to encrypt and decrypt, which converts plaintext into ciphertext [25,26].

**Definition** **3.** *(Symmetric Key Encryption) Symmetric key encryption scheme has the following two algorithms:*

- *Encryption (k,m): The encryption algorithm takes a plaintext message m and a symmetric key k as input and generates the output ciphertext C as a result.*
- *Decryption (C,k): The decryption algorithm takes a ciphertext C and a symmetric key k as input and finally decrypt to the original plaintext message m.*

#### 2.4.3. Identity-Based Proxy Re-Encryption

For further details, we refer to the first construction of [22] for Identity-based Proxy Re-encryption scheme (IB-PRE).

**Definition** **4.** *(Identity-based Proxy Re-Encryption) The following algorithms describe the Identity-based Proxy Re-Encryption scheme:*

*(i)*  *Setup ($1^{\alpha }$): To generate the scheme parameters, it takes a security parameter α as input and outputs the public params and a master secret key msk;*
*(ii)*  *KeyGen (params,msk,id): To extract a decryption key for identity id, it takes the public params,msk and id as input and returns a private key $sk_{id}$;*
*(iii)*  *IBE (params,m,id): To encrypt the message m, it takes the public params, the original message m and identity id and generates the ciphertext $c_{id}$;*
*(iv)*  *ReKeyGen ($params,sk_{id_{i}},id_{j}$): In order to generate re-encryption key from $user_{i}$ to $user_{j}$, it takes input $params,sk_{id_{i}}$ and $id_{j}$ of $user_{j}$ and produces $rk_{id_{i}\to id_{j}}$;*
*(v)*  *ReEnc ($params,rk_{id_{i}\to id_{j}},c_{id_{i}}$): To convert the ciphertext of $user_{i}$ to the ciphertext of $user_{j}$, it takes input $params,rk_{id_{i}\to id_{j}}$ and $c_{id_{i}}$ and gives $c_{id_{j}}$ as output ciphertext;*
*(vi)*  *Decrypt ($params,c_{id_{j}},sk_{id}$): To decrypt the ciphertext, it takes $params,sk_{id}$ and $c_{id_{j}}$ as input and finally generates the original message m as the plaintext.*

*Correctness.* Suppose that all id∈ID and all m∈M message space, the correctness of IB-PRE means that:

(2) $$P_{r}\left[\begin{array}{c} Decrypt(params,c_{id},sk_{id_{i}})=m \end{array}\right]=1,$$

(3) $$P_{r}\left[\begin{array}{c} Decrypt(params,sk_{id_{i}},ReEnc\left(c_{id_{i}},rk_{id_{i}\to id_{j}}\right))=m \end{array}\right]=1$$

## 3. System Overview

In this section, we will describe the system design, security model and assumptions of our proposed scheme.

### 3.1. Overview of the Proposed Scheme

In ES_CD, sensitive contents are encrypted by the content producer using the symmetric key encryption algorithm before disseminated to the network. Additionally, the corresponding symmetric key will be encrypted again by an identity-based proxy re-encryption scheme (IB-PRE) and integrating the time to limit the content access time for each consumer. Each content may be encrypted using the different keys to ensure that only legitimate consumers can obtain the symmetric key to decrypt the requested content. Both the content producer or consumer can be active only at the start of the message exchange stage and do not need to be always online.

Consumers with authenticated identities can get the decryption key to decrypt the content, and then they can attempt the content decryption before the expired time. Apart from that, the edge router re-encrypts the key automatically after the predetermined time. Our proposed scheme ensures the flexible content access control in the case of the legitimate consumer is predefined and registered, and the content producer is already known these consumers in advance.

### 3.2. System Model

**Figure 4:**
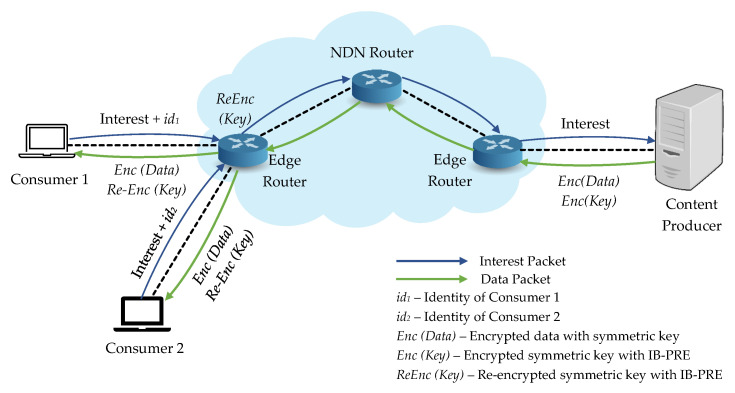
Overview of system architecture for our proposed ES_CD.

We consider the system is composed of the following entities: a content producer, network routers (edge routers and NDN routers), and many consumers, which is shown in Figure 4.

The following are the detailed description for each NDN entity in our work:

- Content Producer: In our system, original data are produced by the content producer and must be encrypted with the symmetric key. Then the corresponding symmetric key is also encrypted with IB-PRE scheme, which can only access and decrypt by the authenticated consumers.
- Consumers: Consumers request the desired content from the content producer and related decryption key. Moreover, only the consumers who can successfully decrypt the encrypted key with their identities can decrypt the encrypted content.
- Edge Router: Edge router is responsible for the re-encryption of the first layer encrypted key into another ciphertext by using the re-encryption key and delivers to the consumers.
- NDN Router: NDN router forwards the requests from consumers to the content producer. It will also cache the data packet before sending it back to the requested consumer and deliver it to other consumers according to the same request.

### 3.3. Set-Up and Assumption

We assume that the semi-trusted edge router which performs the proxy tasks has no key management issues or collusion problem with the consumers. Likewise, the content producer is considered to be a trusted entity and performed the access control processes. Public and private key pairs for each entity are pre-generated, and other entities can easily access the public key. We also believe that the authorized consumers do not store any symmetric key after their decryption process, and there is no collusion between the content producer and the consumers.

### 3.4. Thread Model

We suppose that there are two kinds of adversaries in our proposed system based on our assumptions in Section 3.3:

- Type-I adversary. Unauthorized consumer: Consumers who want to access the sensitive content even though he is not eligible to access and try to send the request packets to the network; and
- Type-II adversary. Compromised revoked consumer: Consumers who have already got the decryption key for the content and been revoked, become malicious and attempt to access the old data with the old successfully decrypted key.

## 4. The Proposed Solution

Our scheme using notations in Table 1 consists of 5 protocols described below (case 1 of Section 4.2). We omit to describe slightly different protocols for case 2 and 3.

### 4.1. Detailed Protocols

*Protocol 1 System Initialization:* Before starting other operations, the system needs to initializes the required public and private parameters for IB-PRE encryption by running Setup(). Let e:G×G→$G_{T}$ be a bilinear map and PKG selects $s\overset{\$}{\leftarrow }Z_{q}^{*}$. The algorithm gives the outputs msk=s which is only shared to the content producer and the consumer and $pk=(G,g,h,H_{1},H_{2})$ to all entities, where $h=g^{s}$ and $G=\langle g\rangle$. Here, $H_{1}$ and $H_{2}$ be the hash functions $H_{1}:{\left\{0,1\right\}}^{*}\to G$ and $H_{2}:G_{T}$→G respectively. Let Enc(.) and Dec(.) be a symmetric encryption algorithm and decryption algorithm, respectively, and IBE(.) be a standard identity-based encryption.

*Protocol 2 Consumer registration:* Every consumer needs to register as an authenticated consumer with their identities before sending the first content request to the content producer. When consumer *i* registers with the identity $id_{i}$, the content producer first authorizes the consumer *i*’s access privilege and returns the registration acknowledgement together with the predefined access time $t_{i}$ to the consumer *i*. Then, the content producer stores the information tuple as $U=(id_{i},t_{i})$ in the authenticated consumer list and shares it with the edge router through a secure channel for further process. As soon as the registration protocol is successful, the consumer can start the content request process by sending an interest packet to the content producer. The registered consumer in the authenticated consumer list does not need to register again for the same content request and it can directly retrieve the cached data until its predefined access time is expired.

*Protocol 3 Secure content distribution:* Upon receiving an interest packet from the consumer, the content producer is responsible for encrypting the original content content for secure content distribution in the NDN network. First, the content producer randomly selects a symmetric key *k*, where $k\in {\left\{0,1\right\}}^{*}$, then it encrypts the original content from the data packet and finally produces the encrypted content Enc(content).

*Protocol 4 Secure Key Distribution:* Before the encrypted content Enc(content) is distributed to the network, the corresponding symmetric key *k* is encrypted by the content producer for secure key delivery to the consumer. This protocol utilizes IB-PRE and performs the following steps:

- KeyGen$\left(pk,msk,id_{p}\right)$: The content producer takes public parameters pk, master secret key msk and identity $id_{p}\in {\left\{0,1\right\}}^{*}$ as input and generates a private key $sk_{id_{p}}=H_{1}{\left(id_{p}\right)}^{msk}$.
- IBE$\left(pk,k,id_{p}\right)$: This step is also performed at the content producer side to encrypt the symmetric key *k* under the content producer’s identity $id_{p}$. It takes symmetric key $k\in G_{T}$ with $id_{p}$ and public parameters pk as input and outputs the ciphertext $c_{id_{p}}=\left(C_{1},C_{2}\right)$. For some random $r\in_{R}Z_{q}^{*}$, it computes $C_{1}=g^{r}$ and $C_{2}=k\cdot e(g^{msk},$$H_{1}\left(id_{p}\right){)}^{r}$.
- ReKeyGen$\left(pk,sk_{id_{p}},id_{i}\right)$: When the content producer wants to delegate his or her decryption right to Consumer *i*, it needs to produce the re-encryption key with the public parameters pk, its private key $sk_{id_{p}}$ and identity $id_{i}$ of the consumer *i* as input. First, it selects a random $X\overset{\$}{\leftarrow }G_{T}$ to perform the encryption $IBE\left(pk,X,id_{i}\right)=\langle R_{1},R_{2}\rangle$. It finally outputs the re-encryption key $rk_{id_{p}\to id_{i}}=\langle R_{1},R_{2},R_{3}\rangle$, where $R_{3}=sk_{id_{p}}^{-1}\cdot H_{2}\left(X\right)$. After that, the content producer returns all together by taking the encrypted content Enc(content), first layer encrypted key $c_{id_{p}}$, and re-encryption key $rk_{id_{p}\to id_{i}}$ to the consumer *i* through the edge router.
- ReEnc$\left(pk,rk_{id_{p}\to id_{i}},c_{id_{p}}\right)$: Before forwarding to the consumer *i*, the edge router first receive the data packet and performs as a proxy. It extracts the re-encryption key part and transforms the first layer encrypted ciphertext $c_{id_{p}}$ to the second layer ciphertext without learning any information about the plaintext of $c_{id_{p}}$. It takes $rk_{id_{p}\to id_{i}}$ and $c_{id_{p}}$ as input and generates a re-encrypted ciphertext:
  $c_{id_{i}}=\langle C_{1}^{\prime }=C_{1},C_{2}^{\prime }=C_{2}\cdot e\left(C_{1},R_{3}\right),C_{3}^{\prime }=R_{1},C_{4}^{\prime }=R_{2}\rangle$. It forwards Enc(content), $c_{id_{i}},rk_{id_{p}\to id_{i}}$ to the consumer *i*.

*Protocol 5 Content Decryption:* After receiving the data packet with encrypted content, re-encrypted symmetric key and the re-encryption key, consumer *i* performs the decryption protocol by using his or her private key $sk_{id_{i}}$. First of all, it starts to decrypt *X* value from $\langle C_{3}^{\prime },C_{4}^{\prime }\rangle$ and then recovers the symmetric key as *k* = $C_{2}^{\prime }/e\left(C_{1}^{\prime },H_{2}\left(X\right)\right)$. Lastly, the consumer *i* decrypts Enc(content) to the original plaintext content with the decrypted symmetric key *k* and the secure content distribution ends.

### 4.2. Secure Content Distribution Process

**Figure 5:**
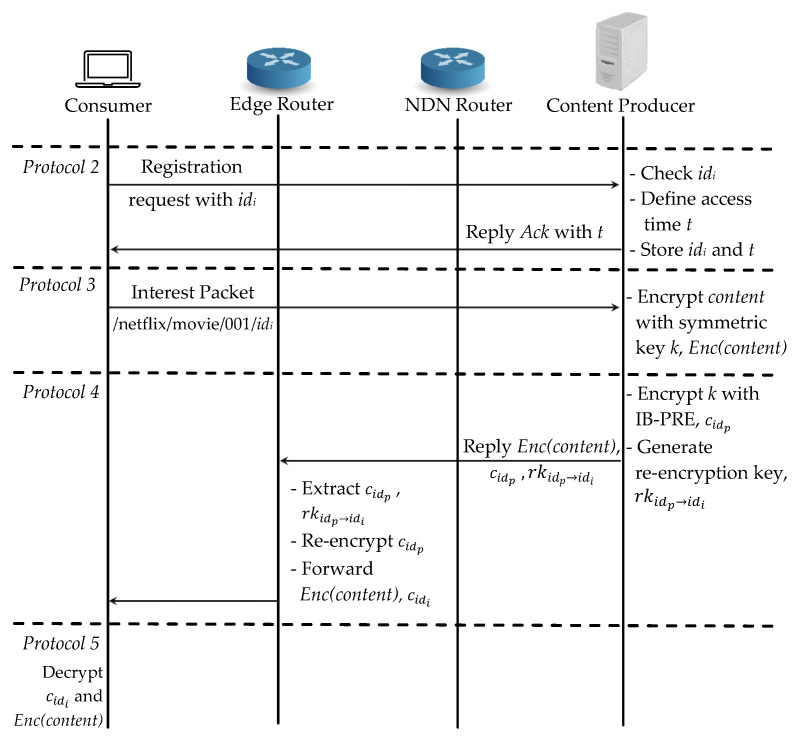
Proposed scheme: Case (1) a consumer requests a fresh content.

There are three cases in our scheme: (1) a consumer requests a fresh content content, which has not been distributed; (2) the same consumer requests the same content content which has already been cached in the router as Enc(content); (3) another consumer requests the same content content. For the case (1), a consumer *i* wants to retrieve content and it needs to register with its identity $id_{i}$ to the authenticated consumer list at the content producer according to *Protocol 2* in Section 4.1. which is shown in Figure 5. As soon as the registration request arrives, the content producer first checks the consumer’s $id_{i}$ and defines a access time *t*. Then it stores the consumer as one of the authenticated consumers and shares the list $\left(id_{i},t\right)$ to the edge router. After registration is finished, the content producer replies the acknowledgement Ack with the valid access time *t* reverse path to the consumer.

Next in *Protocol 3*, the consumer can start the required content request to the content producer. Firstly, it checks the content in the NDN router’s cache; if there is no cache, it will directly request it from the content producer. When the content producer receives the request, it first encrypts the requested content content with symmetric encryption and produces Enc(content). Then the key is encrypted with IB-PRE as $c_{id_{p}}$ in *Protocol 4* and also generates the re-encryption key $rk_{id_{p}\to id_{i}}$ for the edge router to perform a re-encryption operation. It replies Enc(content), $rk_{id_{p}\to id_{i}}$ and $c_{id_{p}}$ to the consumer. When the reply data packet arrives at the edge router, it extracts the re-encryption key and the first layer ciphertext of the symmetric key $c_{id_{p}}$ to re-encrypt $c_{id_{i}}$ and forwards both Enc(content), $c_{id_{i}}$ to the consumer. At last in *Protocol 5*, the consumer can decrypt the symmetric key *k* with its private key and then recover the original content content. All the consumers can access both encrypted content Enc(content) and the first layer encrypted symmetric key $c_{id_{p}}$ from the cache, but the authenticated consumer can only decrypt the re-encrypted symmetric key with its private key.

**Figure 6:**
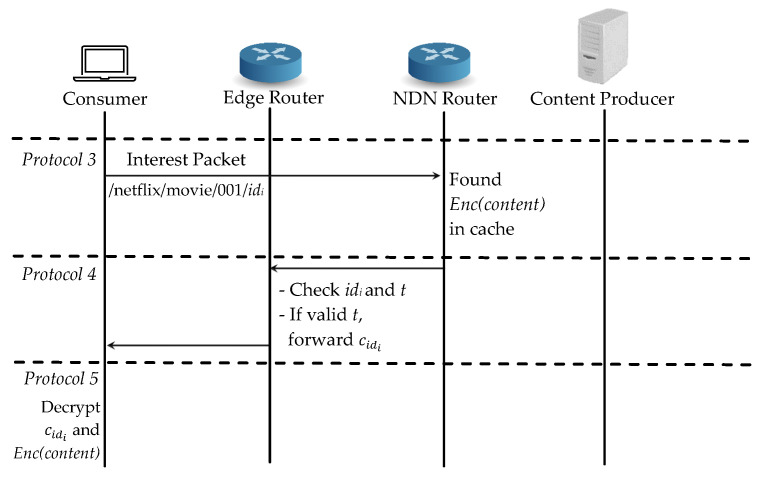
Proposed scheme: Case (2) the same consumer requests the same content.

For the case (2), the same consumer requests the same content again and it can easily retrieve the encrypted content Enc(content) from the cache during *Protocol 2*, as shown in Figure 6. After sending interest packet in *Protocol 3*, it can find the cache data at the intermediate router but the edge router will check if the consumer’s access time is still valid in *Protocol 4*. If the time is valid, the edge router will forward the corresponding re-encrypted symmetric key $c_{id_{i}}$ directly to the consumer. If not, it will revoke the consumer from the authenticated consumer list and the consumer has to start registration from *Protocol 2*.

For the case (3), an another consumer *j* requests the same content content and it needs to register first to the authenticated consumer list at the content producer. Even the Enc(content) is available at the router’s cache, consumer *j* cannot access it if he is not register in the list as an authenticated consumer. First, the consumer *j* registers with its $id_{j}$ and the content producer defines the access time and replies the acknowledgement Ack together with the time *t* in *Protocol 2*.

**Figure 7:**
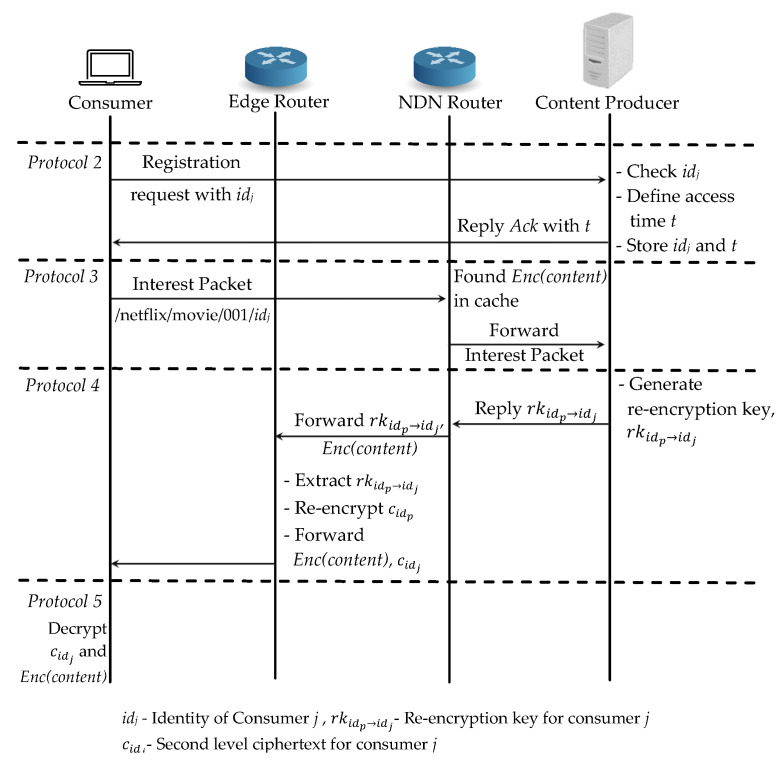
Proposed scheme: Case (3) a different consumer requests the same content.

As soon as the consumer receives Ack, it can sends an interest packet to the content producer. NDN router first checks in the cache and finds the cached encrypted content Enc(content). Then it forwards the interest packet request to the content producer for the re-encryption key generation in *Protocol 3*. When the interest packet arrives at the content producer, it generates a re-encryption key for consumer *j* with $id_{j}$ and replies it to the router in *Protocol 4*. The router then forwards the encrypted content Enc(content) and re-encryption key $rk_{id_{p}\to id_{i}}$ to the edge router for re-encryption process. The edge router extracts the re-encryption key $rk_{id_{p}\to id_{i}}$ and re-encrypts the first layered encrypted key $c_{id_{p}}$ to $c_{id_{j}}$ and then forwards Enc(content) and $c_{id_{j}}$ to consumer *j*. Finally, consumer *j* can decrypt $c_{id_{j}}$ with its private key and then decrypts the original content content in *Protocol 5*. For the same content request from the different consumers, our proposed scheme only needs to re-encrypt the key at the edge router which is shown in Figure 7.

### 4.3. User Revocation

The system performs the user revocation automatically at the edge router by verifying the predefined access time *t* of each consumer to achieve flexible access control for the content producer. When a consumer requests the same content, the edge router checks the incoming consumer’s identity and the access time against the authenticated consumer list shared by the content producer as given in Table 2.

Then it will share the re-encrypted decryption key only to the consumer whose predefined access time is not expired. When the consumer’s predefined access time is expired, the re-encryption key will be re-generated and the first layer encrypted symmetric key needs to be re-encrypted. Afterwards, this consumer will be revoked from the system and removed from the authenticated consumers list. Specifically, the content producer and the edge router need regular contact for updating the registration list to perform the scalable user revocation.

## 5. System Evaluation

### 5.1. Security Analysis

The original content is securely encrypted with the symmetric key, randomly generated only by the content producer. Then the corresponding key is encrypted with IB-PRE for secure key transmission only to the authorized consumers. We implement IB-PRE on an elliptic curve using 224-bit group order, and IB-PRE itself is secure under the Decisional Bilinear Diffie-Hellman Assumption (DBDH) in the random oracle model. IB-PRE also supports non-transitivity and unidirectional so that the consumer does not need to share its secret key or participate in the re-encryption process. It ensures that even the edge router is compromised, it cannot decrypt the re-encrypted key or the encrypted content since it does not have the consumer’s secret key.

Moreover, the content producer has full access authority on its content; only registered consumers can decrypt the encrypted content. It has the access right to block all unauthorized registrations and will not reply to the registration acknowledgement message and maintains the authorized consumer list. Assume that an unauthorized consumer (Type-I adversary) wants to request the cached content in the router; the edge router will not generate the re-encrypted ciphertext since this consumer is not in the pre-registered list. Even though it can access Enc(Content) and the first layer ciphertext of decryption key $c_{id_{p}}$ from the cache, it cannot recover the decryption key to get the original content which is securely encrypted with IB-PRE.

In the case that a revoked consumer (Type-II adversary) becomes curious and requests the content beyond its access time and there is no previous downloaded content, the edge router will hinder the re-encryption operation and automatically execute the user revocation so that it will need to register again to the content producer and get the new decryption key. Accordingly, malicious consumers cannot learn any information about the encrypted data, and content confidentiality for both fresh contents and cached contents can be guaranteed in our proposed system. In the case that the revoked consumer has already downloaded the encrypted content Enc(content) and the re-encrypted key $c_{id_{i}}$, it can decrypt $c_{id_{i}}$ to retrieve the decryption key *k* and access the content with the key *k*. The revocation of our scheme does not prohibit the access to the contents already downloaded but prohibits the access to newly encrypted contents.

### 5.2. Performance Analysis

#### 5.2.1. Computational Overhead

All the statistical analyses for cryptographic performance were conducted on different numbers of VMs with Ubuntu 18.04 LTS running on an Intel(R) Core (TM) i7-8700 CPU @ 3.20 GHz and 8 GB of Memory. We utilize a 128-bit AES in cipher-block-chaining mode (AES-CBC) as the symmetric encryption algorithm, one of the default content-encryption algorithms described in [13]. We implement the IB-PRE algorithm for secure distribution of symmetric key, the first construction of [22]. To implement the cryptographic algorithms, we use the encryption libraries, such as GNU multiple precision arithmetic (GMP) [27] and pairing-based cryptography (PBC) [28] with the development language C++. For NDN operation, we employ NDN software and libraries [29] to forward and receive interest and data packets. We present various analyses for both theoretical and experimental, showing that our proposed system is practical and efficient to apply in NDN in the following section.

In our implementation, the original content is securely encrypted with a randomly selected symmetric key at the content producer. The corresponding symmetric key *k* is then encrypted with IB-PRE, which is implemented using the parameters from Type A pairing with the group order 224-bit on a secp224k1 elliptic curve. We set up a compact NDN environment with one content producer, one consumer, one intermediate router, and one edge router to evaluate the computational overhead of our system.

The consumer requests for a 2 KB content file to the content producer by sending the interest request as /netflix/movie/001. Then the content producer performs content encryption, corresponding key encryption, and re-encryption key generation and appends all the keys along with the encrypted data and returns it to the consumer in a data packet as /netflix/movie/001/enc_data/enc_key/RK/. The edge route first extracts /enc_key/RK/ part from the data packet to perform re-encryption. It re-appends to the data packet as /netflix/movie/001/enc_data/reenc_key/ and forwards it to the consumer. Finally, the consumers can get the symmetric key by decrypting the re-encrypted key, and then the requested content can be recovered.

We execute the simulation by running it 30 times repeatedly by requesting the same file size by the consumer. Then we analyze the average computational performance for each cryptographic operation at the content producer, consumer, and edge router, presented in Table 3. The results demonstrate that IB-PRE key generation and re-encryption key generation perform at a high cost than others with 4.461 ms and 5.272 ms which needs to perform only once at the content producer. As for IB-PRE encryption and decryption only take mostly 3 ms, while re-encryption takes less than 1ms at the edge router.

**Figure 8:**
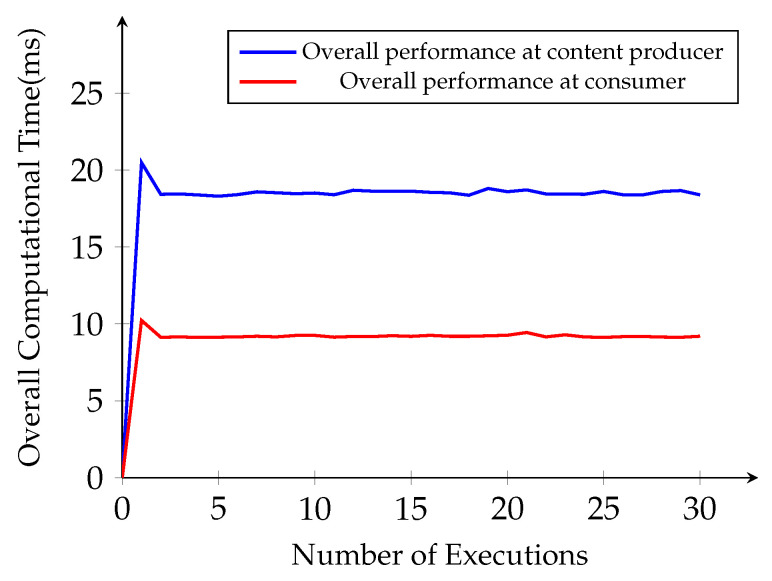
Average overall computational time.

Then, we investigate the total time complexity from the same simulation for content encryption and decryption operations at the content producer, and the consumer side starts from the content request until decryption of the original content. After a number of executions, the overall time becomes linear as the number of implementation executions for the same size content with 19 ms at the content producer side and 9 ms at the consumer side, as plotted in Figure 8. For the content, symmetric encryption and decryption perform efficiently with the faster computation time, 0.226 ms for encryption and 0.199 ms for decryption without any significant overhead for NDN end entities.

**Figure 9:**
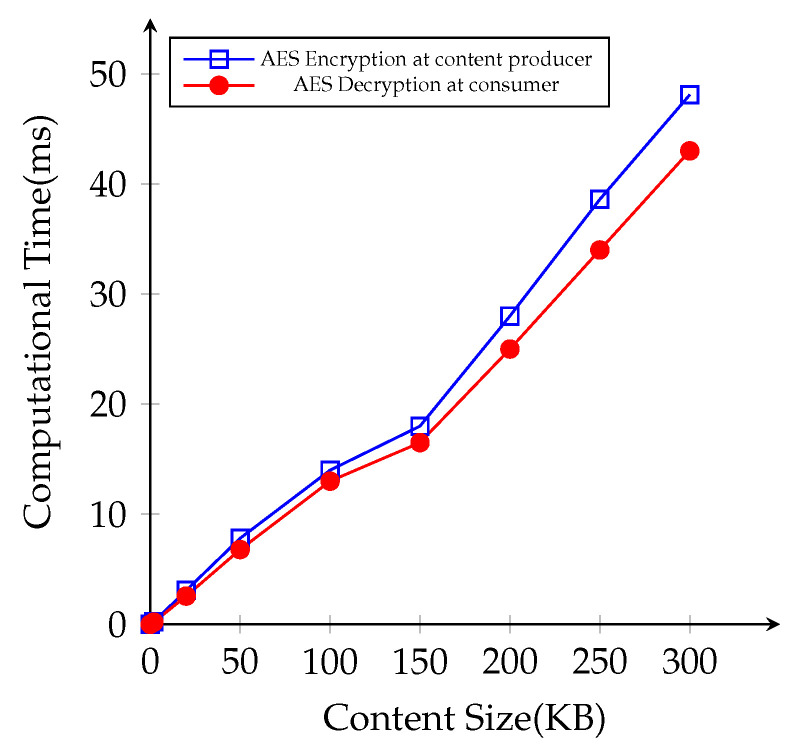
Average computational time cost for AES operations with different file sizes.

We conduct another simulation with different file sizes in the same NDN environment. We prepare the content file sizes with 2 KB, 10 KB, 50 KB, 100 KB, 150 KB, 200 KB, 250 KB, and 300 KB at the content producer, and the consumer attempts to request different file to examine the impact of content file sizes on cryptographic operations. First, we measure the time cost for AES encryption for the original contents at the content producer side and AES decryption time at the consumer side, respectively, as illustrated in Figure 9. The consumers send successive content requests to finish downloading the whole file completely. In fact, the results were statistically significant that AES performance time gradually increases with respect to the content file sizes.

**Figure 10:**
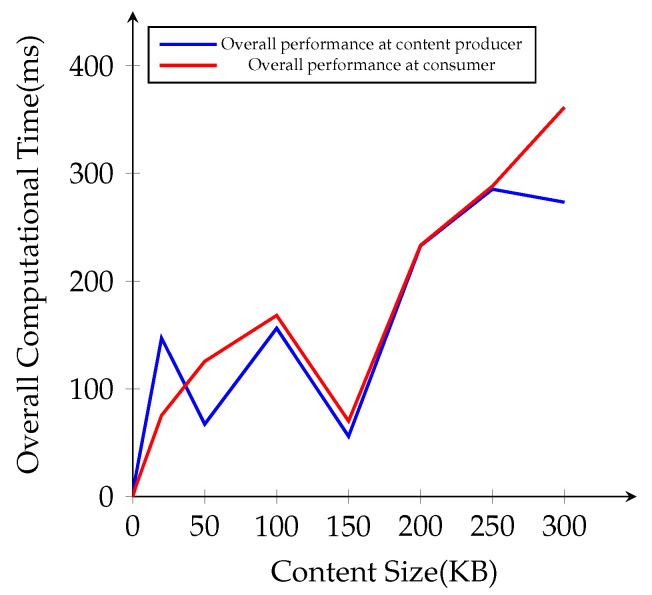
Overall computational time cost for different file sizes.

The overall cryptographic performance time with different file sizes at the content producer and the consumer is presented in Figure 10. Compared to AES content encryption and decryption process, IB-PRE operation times are not related to the content file sizes, and sometimes the overall time cost becomes lower for larger file sizes in 50 KB and 150 KB. Hence, we apply IB-PRE only to encrypt the symmetric key *k*, which has the same size for every content, not to directly every original content. When we apply our scheme to the NDN environment in the simulation, it performs well with lower computational overhead for small file sizes. Overall, our scheme takes more computational time for AES content encryption and decryption for the larger file sizes.

#### 5.2.2. Communication Overhead

**Figure 11:**
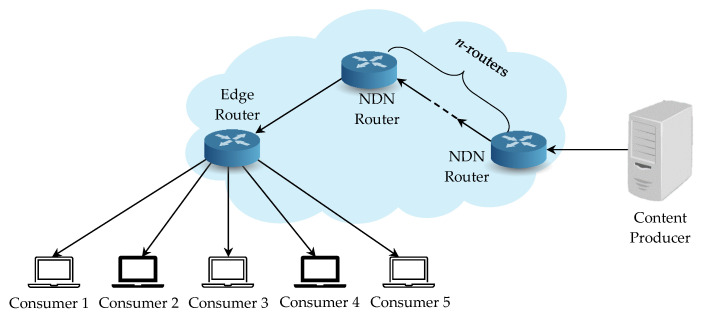
Network topology for performance analysis.

To examine the performance analysis for network communication cost, we set up an experimental environment by constructing a different number of VMs with 2 CPU and 2048 MB of Memory to simulate the NDN entities. All the VMs are running on an Intel(R) Core (TM) i7-8700 CPU @ 3.20 GHz and 8 GB of Memory. We use the tree topology in the network simulations, as illustrated in Figure 11, with one content producer, one edge router, *n* NDN routers, and five consumers. Each NDN entity is connected through the average bandwidth 200 Mbps and 5 ms network delay. Both the NDN router and the edge router adopt the NDN’s default First In First Out (FIFO) caching policy to replace the oldest cached contents with the new incoming contents and forward with the best route strategy. The cache size in content store is limited by the number of packets, and the default is 65,536 packets so that 500 MB data can be stored in the cache in total.

**Figure 12:**
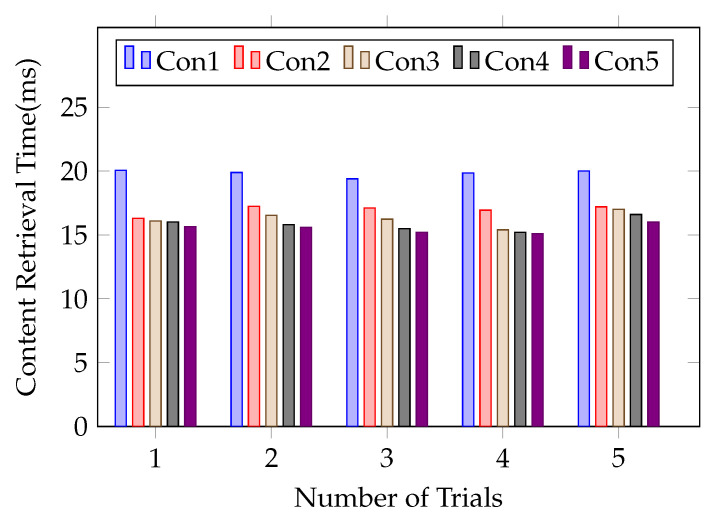
Content retrieval delay for different number of consumers with different identities.

We conduct the network simulation and compare the content retrieval time for both fresh and cached content for 5 different consumers which perform successive requests, as shown in Figure 12, where Con1,…, Con5 denote different consumers, each consumer sends the same content request with 2 KB size and the number *n* of routers is 1. We run the experiment five times and analyze the cached content retrieval for different consumers. In our simulation, a consumer needs to obtain both encrypted content and encrypted key directly from the content producer for the first content request cached in the router, and the content retrieval process may take more time. When revocation occurs, we do not need to re-encrypt and re-publish the original content, and it only needs to perform the content decryption key re-encryption at the edge router at an acceptable cost and lower communication overhead in the NDN environment. Figure 12 shows that the content retrieval time is reduced for the cached content by 10% to 25% on average.

**Figure 13:**
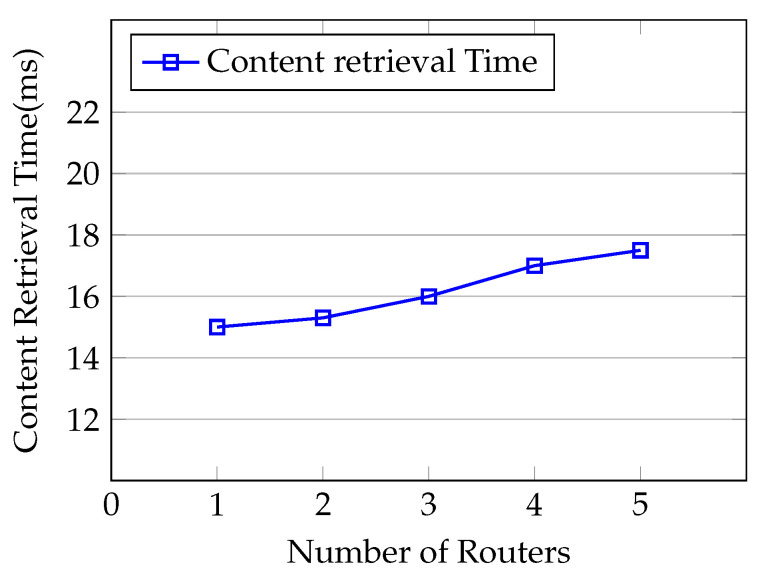
Content retrieval delay for n-number of routers.

Finally, we measure the effects of adding many routers in our simulation scenario to highlight the impact of communication overhead on the consumer to retrieve the content. We set up the experiment where one consumer requests the 2 KB content from the content producer and we increase the number of intermediate routers, i.e., n=1,2,…5, between the content producer and the consumer in each simulation. As shown in Figure 13, we can clearly see that the content retrieval time for the consumer slightly increases with the number of routers in the network. The consumer needs to pass through *n* numbers of routers to register at the content producer, requests the content and retrieves the content.

## 6. Comparisons

We pay attention to the schemes [30,31,32,33] having the similar features as our proposed scheme, i.e., use of hybrid encryption, no need of trusted third party, and effective use of in-network caching mechanism. We compare our scheme with the schemes with respect to the various factors associated with the encryption-based access control in NDN, as described in Table 4. Compared to [30], where each consumer has to obtain the content decryption key directly from the content producer so that the consumers have to always contact the content producer, and the content producer needs to be online all the time, which incurs additional content retrieval delay. For each key request, the content producer must first validate the consumer access right and sends the key. The key retrieval process entirely depends on the content producer. If the content producer becomes offline, content accessibility, and availability diminish. In our work, we add the edge router to perform as a semi-trusted proxy for the re-encryption process. The consumer can access the decryption key directly from the edge router and no need to contact the content producer. The content producer can be online only at the content distribution and re-encryption key generation process. Later, it can be mostly offline until the consumer becomes revoked and registers again to the system that can significantly reduce the content retrieval time and communication costs between the content producer and the consumer in NDN.

Although, the scheme [31], provides the cache awareness in the same level as our work, it incurs higher storage and computational burden in the network since it needs to store the key management tables at the edge router. When an interest request arrives, it needs to check against both tables for further message flows and key management operations. In ES_CD, we only need to store the authenticated consumer lists at the edge router which is shared by the content producer. Then the edge router only needs to check the consumer’s identity with access time which incur only a slight computational cost in the network.

ES_CD maintains the in-network caching ability of NDN for every consumer who has registered at the content producer as an authenticated consumer. For the same content requests by the consumer, Enc(content) is easily accessible from the router’s cache. Furthermore, key re-encryption and user revocation happen at the edge router so that there is no significant content retrieval delay caused by contacting the content producer. Concerning the computational cost, our scheme incurs an extra cost only at the content producer but not at other entities since the content producer performs AES encryption and re-encryption key generation. Our analysis shows that the average computational time for the 2 KB content is less than 19 ms at the content producer side and 9 ms at the consumer side, respectively. Furthermore, the computational time for the router is smaller than the content producer since the router only needs to forward and reply the packets without any computation. Thus, the consumer and the router in our scheme are free from high computational overhead compared to [31,32,33] and similar to [30], as shown in Table 4.

Although all other schemes [30,31,32,33] fulfill the usage of in-network caching in parallel, our scheme provides content retrieval time reduction with 10% to 25% for cached content since the encrypted content is available for all consumers from the router’s cache and only key re-encryption is required at the edge router. To sum up, ES_CD provides comparable properties for content confidentiality with others schemes and guarantees effective cache utilization for every content. Our scheme fits well with the NDN architecture with an acceptable computational and communication overhead without the need for an additional trusted third party.

## 7. Related Work

We further examine the prior works on encryption-based access control for secure content distribution and data privacy. Even NDN packets do not contain any information about the source host where they were originally sent; contents security and privacy are still needed. Different cryptographic approaches have been explored in access control solutions for NDN, such as attribute-based encryption, broadcast encryption [32,33], role-based encryption, identity-based encryption [30,34], proxy re-encryption [31,35,36], and so on [7,37,38]. These approaches are examined below. The key problem of [30,31,34,35,39] these schemes are the computational and communication overhead for the content producer and the consumer, while other schemes [32,33,36,40] do not consider user or key revocation.

A flexible content security scheme is designed in [30] by combining identity-based and proxy re-encryption schemes for content access control in content-centric network (CCN). The original contents are encrypted with the symmetric keys, and the consumers can request the decryption keys upon receiving the encrypted contents. The intermediate node re-encrypts the symmetric keys, and the re-encryption keys are sent back to the consumers. Even though their scheme can utilize in-network caching to retrieve the content, consumers must contact the content producer for decryption key requests individually. So, the content producer must always be online for the re-encryption key generation, which creates an additional burden. The delay of key delivering and issuing hinders content availability for each consumer. Additionally, the scheme does not consider how user and key revocation is done.

An in-device PRE scheme is introduced to accelerate a flexible access control where a user can easily manage the access policy with proxy attached to the devices themselves in [31]. This scheme also adds some modifications to NDN names as an additional structure /AC/AppID/CN/RK/Nonce/ where AC is the access control structure, AppID is the application identifier, CN is the content name, RK is the re-encryption key. The proxy maintains a pending name table (PNT) which records the content name and its AppID, and a re-encryption key table (RKT) which stores the re-encryption key RK. RTK is used to perform re-encryption processes in the user devices for effective key management and revoking access to the content. Even though this scheme can utilize the in-network caching ability of NDN, the proxy needs to maintain and manage the tables PNT and RKT for managing keys which can incur computational and storage overhead for the proxy as well as in the network.

An authenticated re-encryption scheme that provides sender authentication and data confidentiality with a low storage cost is proposed in [34]. This scheme focuses and guarantees content protection but it cannot provide sufficient performance and also it does not consider the revocation mechanism as [30]. The authors consider an edge re-encryption access control scheme [35] by utilizing the PRE scheme, in which the content producer encrypts the content. Then, the key is also encrypted by the content producer and re-encrypted again by the edge router to ensure that the users can retrieve the cached contents directly from the router. However, the proposed system performs revocation directly by the content producer and then notifies the edge router. The content producer needs to be always online, and it can suffer from communication and computational overhead at the content producer side.

Nikos Fotiou and George C. Polyzos [39] propose to apply PRE to the ICN framework to limit the access control to the contents and provide content confidentiality. They allow the semi-trusted proxies to re-encrypt the contents available only for authorized users. Every user maintains their own public key generator to protect a key escrow problem, but it leads to the overhead of extra storage cost at the user side. Moreover, it also needs the authentication and secure channel between the content producer and the consumer to transfer the messages. A primary limitation of this research is that the contact to the content producer is required for the authentication of consumer in every content request, which creates a significant delay for users to get the contents.

The paper [32] presents a lightweight and secure edge-side access control scheme in ICN where the edge routers perform authentication based on each users’ signatures. It only allows authenticated requests to enter the network, and the content confidentiality is ensured by encrypting contents with broadcast encryption at the producer side. Another work with broadcast encryption is introduced in [33] by combining time tokens for content access control limitation in ICN architecture. Before sending user’s request to the content producer, the edge router performs the user verification process, and which induces extra computational overhead on network. Besides, compare to our scheme, both schemes [32,33] have no consideration for user and key revocation.

In 2015, a scheme built on the efficient unidirectional proxy re-encryption [36] is proposed in which the user performs the proxy task for the re-encryption process without adding the edge router. The scheme needs an assumption that the users are semi-trusted entities. Limitations to the study design include key management overhead and user revocation. Chen et al. explore novel encryption and probabilistic-based access control scheme [40] based on bloom filter for the NDN video streaming application. Whereas it can give end-to-end content security, using bloom filter to store the user’s public key can increase memory consumption and lead to false positives.

Although there have been various studies and techniques in NDN access control, further systematic and theoretical analysis concerning content security and better performance are still needed. In general, almost all the present encryption-based access control schemes suffer from computational and communication burden between entities for the content and decryption key request. In addition to this, some studies constrain cache utility, and only a few works consider the revocation mechanism.

## 8. Conclusions

We proposed an efficient and secure content distribution scheme (ES_CD) by employing symmetric encryption, identity-based proxy re-encryption, and limiting access time to assure our design goal of content security for sensitive contents and protect content leakage in NDN. Our work provides flexible access control in distributing content through the network so that the content producer can take advantage of its control over the cached contents. ES_CD incurs lower computational and communication overhead since it needs to modify the decryption key only, not the entire content for re-encryption or redistribution to the network. Finally, our analysis reveals that ES_CD is suitable for the application to the NDN architecture with acceptable cost and still utilizing the NDN in-network caching feature. Therefore, in future research we should employ a large scale realistic NDN environment and explore more about cached content privacy and security issues.

## Figures and Tables

**Table 1:** List of notations used in our scheme.

Notation	Description
msk	Master secret key
pk	Public parameters
PKG	Private key generator
id	Identity
$id_{p}$	Identity of the content producer
$id_{i}$	Identity of Consumer *i*
*U*	Identity and access time tuple
$t_{i}$	Predefined access time for Consumer *i*
content	Original content
*k*	Symmetric key
Enc(content)	Encrypted content
sk	Secret key
$c_{id_{p}}$	First level ciphertext
$sk_{id_{p}}$	Content producer’s secret key
$sk_{id_{i}}$	Consumer *i*’s secret key
$rk_{id_{p}\to id_{i}}$	Re-encryption key
$c_{id_{i}}$	Second level ciphertext

**Table 2:** Authenticated consumer list.

Identity	Expiration Time
$id_{1}$	$t_{1}$
$id_{2}$	$t_{2}$
$id_{3}$	$t_{3}$
…	…
$id_{n}$	$t_{n}$

Where $t_{1}$, $t_{2}$, $t_{3}$,…, $t_{n}$ can be daily, monthly or yearly based on consumer’s registered identity.

**Table 3:** Computational Time for each cryptographic operation.

Operation	Time (ms)
AES Encryption	0.226
Pairing Generation	1.754
Key Generation	4.461
IB-PRE Encryption	3.595
Re-encryption Key Generation	5.272
Re-encryption	0.801
Consumer Key Extraction	2.627
IB-PRE Decryption	3.171
AES Decryption	0.199

**Table 4:** Comparison with related access control mechanisms.

Scheme	CO			Content Confidentiality	Cache Awareness	Offline CP	AC Approach	Revocation
	CP	Con	R					
Flexible [30]	H	L	L	Yes	Yes	No	PRE, IBE	Not considered
LASA [32]	H	H	L	Yes	Yes	No	Broadcast encryption	Not considered
TSLS [33]	H	H	L	Yes	Yes	No	Broadcast encryption	Not considered
In-device [31]	H	H	L	Yes	Yes	No	PRE and IBE	Lazy revocation
Ours	H	L	L	Yes	Yes	Mostly	Symmetric, IB-PRE	Automatic revocation

CO—Computational Overhead, CP—Content Producer, Con—Consumer, R—Router, AC—Access Control, H—High, L—Low.

## Abbreviations

The following abbreviations are used in this manuscript:

NDN	Named Data Networking
ICN	Information-Centric Networking
CCN	Content-centric Networking
ES_CD	Efficient Secure Content Distribution
PRE	Proxy Re-encryption
IBE	Identity-based Encryption
IB-PRE	Identity-based Proxy Re-encryption
AA	Access Authority
TA	Trusted Authority
CS	Content Store
PIT	Pending Interest Table
FIB	Forward Information Base
DBDH	Decisional Bilinear Diffie-Hellman
id	Identity
AES-CBC	Advanced Encryption Standard-Cipher Block Chaining
GMP	GNU Multiple Precision Arithmetic
PBC	Pairing-based Cryptography
VM	Virtual Machine
CCA	Chosen-Ciphertext Attack
Con	Consumer
PNT	Pending Name Table
RKT	Re-encryption Key Table
FIFO	First In First Out
